# Towards a self tuning sliding mass metastructure

**DOI:** 10.1038/s41598-021-00526-w

**Published:** 2021-11-03

**Authors:** Mohammad A. Bukhari, Oumar R. Barry

**Affiliations:** Mechanical Engineering, Virginia Tech, Blacksburg, 24060 USA

**Keywords:** Mechanical engineering, Structural materials

## Abstract

Passive vibration control systems are characterized by their simple practical design and independence of external power supplies. However, they are usually hindered by their narrow frequency band that cannot handle variable frequency disturbances. Recent research has demonstrated the capability of passive self-tuning resonators through the use of a sliding mass without the need for any external power sources. This work analytically and experimentally investigates the passive self-tuning of a metastructure consisting of a clamped-clamped beam with a sliding mass. The governing equations of motion show that the slider can be driven by Coriolis and centrifugal forces upon applying the excitation force on the structure. To improve the accuracy of our analytical simulations, we derive the exact instantaneous mode shapes and frequencies of the structure and feed them into an adaptive algorithm, which updates the spatial state of the system. Numerical simulations demonstrate that the proposed resonator can tune itself to the excitation frequency as the slider reaches the equilibrium position. This observation suggests that a significant vibration reduction can be obtained using the proposed resonator over a wide frequency band. Experiments are carried out to validate the analytical findings. The proposed structure can be used in different vibration control applications (i.e., aerospace, automotive, and machining), and its model can further be extended to self-adaptive periodic structures (metamaterials).

## Introduction

Many engineering structures are susceptible to unwanted vibrations from different environmental sources and disturbances. These vibrations can lead to different types of failures if left uncontrolled. These failures may result in structural damage, system malfunction, discomfort, and noise problems. These unwanted vibrations can be mitigated using various passive and active techniques. Linear passive vibration resonators have been extensively investigated and are widely employed in many engineering applications due to their low cost and simple design^1,2^. Passive vibration resonators are tuned spring-mass systems that are installed on a structure to absorb undesired vibrations. To do so, the vibration resonator parameters are chosen so that the resonator will tune itself to a specific frequency, leading to vibration mitigation of the primary structure.

Since linear vibration resonators can be effective only near their design frequency, they fail to control vibrations that are not constant over time. Therefore, introducing mechanisms which can resonate at a wider range of frequencies has been the focus of researchers for many years. It has been observed that mechanisms with nonlinear resonators are more suitable for such purposes due to their wide bandwidth compared to linear resonators and the presence of secondary resonances^3–8^. However, the improvement from nonlinear passive mechanisms is limited to a certain frequency regions, and it is often dependent on the initial disturbance to track the required energy orbit^9^.

To overcome the narrow frequency band of passive resonators, researchers suggested the use of active frequency tuning techniques capable of adapting to the applied excitation frequency. However, due to the required external energy source, active tuning techniques are not practical in most applications^10^. On the other hand, passive self-tuning mechanisms showed the ability to tune themselves to the applied excitation frequency. This self-tuning can be achieved through a sliding mass which moves freely along a beam. Particularly, Coriolis and centrifugal forces, resulting from the nonlinear interactions between the slider and the vibrating beam, move the mass till it reaches an equilibrium position. At this position, the resonator will be tuned to the excitation frequency and hence, achieving passive self-adaptive tuning. The study of these systems revealed substantial improvement in increasing the resonator’s operating bandwidth in several applications including energy harvesting^11–29^.

**Figure 1:**
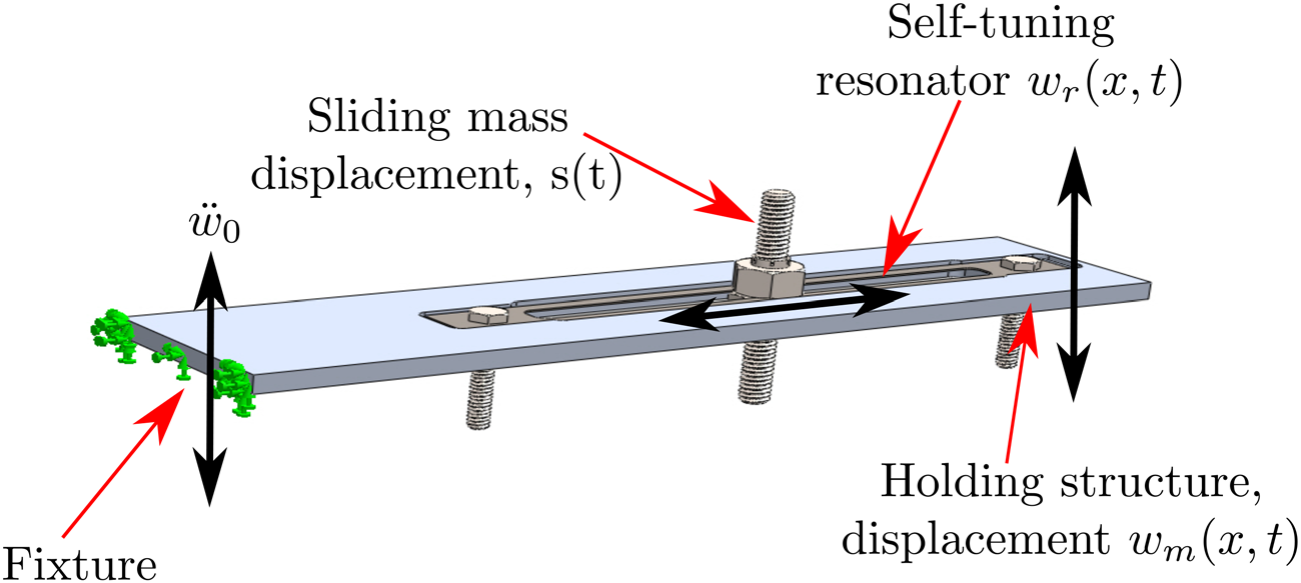
Key components of the self-tuning vibration absorber installed on a structure.

**Table 1:** System key parameters.

Parameter	Value	Unit	Parameter	Value	Unit
*a*	0.1397	m	$\rho _r$	7860	kg/$\hbox {m}^3$
$t_m$	0.0032	m	$\rho _m$	2700	kg/$\hbox {m}^3$
$w_m$	0.0318	m	$E_m$	68.9	GPa
$a_r$	0.0746	m	$E_r$	207	GPa
$t_r$	0.3048	mm	$x_1$	0.0494	m
$w_r$	9.5	mm	$x_2$	0.0156	m
*M*	7.4	g	$w_{m2}$	0.0103	m

Investigations of energy harvesters with passive self-tuning mechanisms have recently drawn researchers’ attention. Analytical investigations of self-tuning resonators’ models can be found in the literature for strings^27^, beams^20,21,25^, and two-dimensional plates^28^. These analytical findings were further verified experimentally in several studies^12,13,16,21–23,25^. Interestingly, investigations of beam structures have shown the capability to obtain self-tuning by a slider regardless of the boundary conditions. For instance, a clamped-clamped system was investigated by Miller et al. and others^11,16,21–23^, while a free-end system was studied by Mori et al. and others^12,13,25,26^. The nonlinear dynamics of the system have also been investigated in-depth to reveal a further understanding of the system by Krack et al.^19^. These interesting findings motivated other researchers to extend the system in an array of several cantilever beams with sliders^14,15,17^. We emphasize that previous studies have reported the dynamic of self-tuning mechanisms with sliding mass for the resonator itself^20,25^, electromechanical energy harvesters^21^, and electromagnetic energy harvesters^30^. However, to the best of our knowledge, there is no work in the literature which investigates the interaction between a passive self-tuning resonator and primary structure and studies how this resonator acts as a broadband vibration absorber. This tunable absorber can further be used in other engineering applications like tunable metastructures and metamaterials. This is the focus of the current study. Given that the dynamic parameters of the system change as the slider moves along the resonator, the system dynamics evolve both in the spatial and the time domains. To handle these changes in both time and spatial domains for first time, our current work also presents an adaptive algorithm to simulate the system dynamics by updating the spatial state as the system is being integrated over time.

In this paper, we investigate the performance of a self-tuning absorber installed on a structure. The self-tunability is targeted to be achieved through a sliding mass which moves along the resonator. We present the nonlinear governing equations of motion for the slider, resonator, and main structure, then obtain the exact linear frequency equation and the mode shapes of the continuous system. These mode shapes and linear frequencies are further used to discretize the partial differential equation using Galerkin’s projection. Unlike conventional methods, we present an algorithm to feed the instantaneous system frequency in the spatial parameters to obtain more accurate results and guarantee the convergence of the slider to an equilibrium point. This algorithm is used to integrate the system numerically to solve for the system response and the slider position, thus investigating the effect of self-tuning absorber on the system response. Parametric studies are also conducted to investigate the role of different parameters on the system response. Furthermore, the proposed system is examined experimentally to demonstrate the ability of the system to passively tune itself to the excitation frequency.

## Results

### System description

A schematic of the primary structure along with the self-tuning resonator is depicted in Fig. 1. The primary structure is chosen to be a cantilever beam which is excited at its fixed end by a base excitation acceleration $\ddot{{\bar{w}}}_0$. The cantilever beam has a length of *a*, thickness $t_m$, width $W_m$, density $\rho _m$, and modulus of elasticity $E_m$. Embedded within the primary structure is a self-tuning resonator to mitigate the system resonance peaks. This resonator consists of a clamped-clamped beam with the length of $a_r$, thickness $t_r$, width $W_r$, density $\rho _r$ and modulus of elasticity $E_r$. The self-tuning in the resonator is achieved by a sliding mass, attached to the fixed-fixed beam (as shown in Fig. 1), with a total mass of *M*. This mass is free to slide along the resonator beam due to Coriolis and centrifugal forces to tune the resonator. A full derivation for the system governing equation can be found in the supplementary material. The parameters of the simulated structure are shown in Table 1, where the primary structure is made from aluminum alloy while the resonator is made from spring steel.

**Figure 2:**
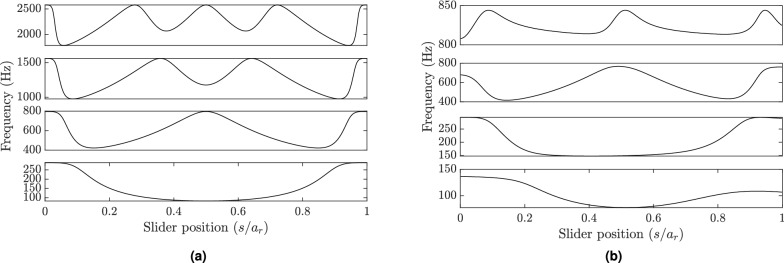
The lowest four resonance frequencies at different slider positions: (**a**) Slider beam only; (**b**) combined system.

#### Resonator and system linear frequencies

Based on the parameters listed in Table 1, we determine the resonance frequencies of the resonator beam with clamped-clamped ends and show the results in Fig. 2a for different slider positions. From Fig. 2a, we can observe that the resonator beam can exhibit a wide range of frequencies at different vibration modes for different slider positions. For instance, it ranges from 82.2 to 288.8 Hz at the first vibration mode for different slider positions (as shown in Fig. 2a). This observation further implies that the resonator can handle a wide range of frequency bands unlike conventional tuned spring-mass systems. Moreover, the current model of the resonator also covers a wide frequency range at higher modes (as shown in Fig 2a), while the gaps between the modes’ resonance frequency bands is narrow. This also indicates that the proposed resonator can be operated at a wider range of frequencies to cancel higher resonance peaks. It is noteworthy that the resonator has multiple potential equilibrium points at each point excluding frequencies at the local minima and maxima of the curve. These equilibrium positions depend on the initial conditions and the boundary condition of the resonator. The number of equilibrium positions for the clamped-clamped resonator could be up to twice the $n^{th}$ resonance mode. For instance, for the third vibration mode, there are six equilibrium positions, as shown in Fig. 2a. Again, we neglected the slot inside the resonator beam in our simulations to simplify our model since our goal is to prove the self-tunability concept of our structure and record the achieved broadband vibration attenuation.

**Figure 3:**
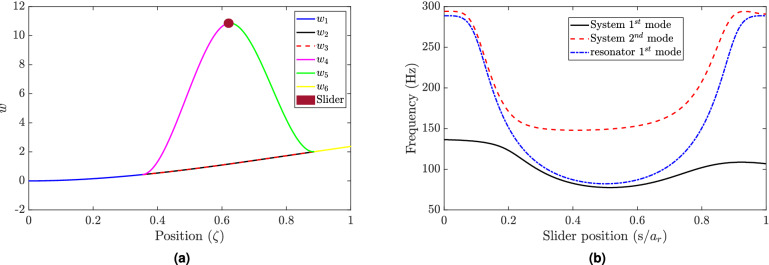
(**a**) The first mode of the combined structure at slider position $s=0.5a_r$; different systems’ portions highlighted in the supplementary material (i.e., $w_i$ are demonstrated in the legend in addition to the slider); however, the y-axis represents the structure displacement (i.e., *w*); (**b**) comparison between the combined structure mode shapes and the resonator at different slider positions.

Next, we investigate the resonance frequencies for the whole structure (i.e., the cantilever beam with the self-tuning resonator). The frequencies corresponding to the first four modes of the combined structure are shown in Fig. 2b for all possible slider positions. These results demonstrate the significant dependency of the structure’s resonance frequencies on the slider position. For instance, the first vibration mode of the structure ranges from 77.5 to 136.4 Hz, as depicted in Fig. 2b. This variance in the structure’s resonance frequency may stretch over several hundred Hz at some vibration modes, as shown in Fig. 2b. These results also show the presence of multiple equilibrium positions at some frequency values. However, the slider will track the equilibrium positions of the single resonator to tune it as discussed below. Note that the frequency curve is not symmetric about the middle point of the resonator beam as the primary structure has different boundary conditions at each end, and the resonator’s center point does not coincide with the center point of the structure. Therefore, the system may exhibit more equilibrium points at lower vibration modes than higher modes, as shown in Fig. 2b. An example of the mode shape is shown in Fig. 3a for the first vibration mode corresponding to a specific slider position, $s=0.5 a_r$. At this position, the system has the minimum resonance frequency in the first vibration mode, as shown in Fig. 2b. Although the combined structure mode shape appears to have the first mode shape of a cantilever beam, the resonator mode shape seems to be different from the primary structure mode shape and has a vibration amplitude higher than any part of the primary structure.

Finally, we plot the resonance frequencies of the first and the second vibration modes of the combined structure and compare it with the first vibration mode of the resonator for different positions of the slider, as shown in Fig. 3b. The result shows that the resonator resonance frequencies lie between the first and second vibration mode frequencies of the structure. Indeed, the presence of the resonator can lead to a split in the first resonance peak of the bare structure (i.e, without the resonator) into the two modes as shown in Fig. 3b. In addition, it can be observed that the resonance frequency curve of the resonator does not intersect with the resonance frequency curves of the structure. Therefore, if the resonator is tuned to the excitation frequency and the slider reaches the equilibrium position, the structure will not go under resonance as both resonance modes are away from the excitation frequency at this equilibrium position.

**Figure 4:**
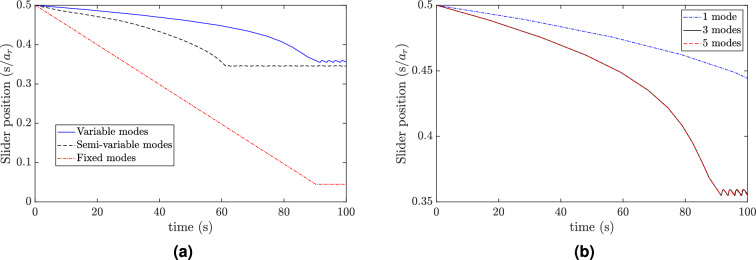
(**a**) Comparison of equilibrium position of slider using different algorithms; Variable modes: updating the mode shapes at every time step, semi-variable modes: updating the modes at a larger time step, fixed modes: using assumed fixed mode shapes. Other parameters for simulation are $s_0=0.5/a_r$, $f=90$ Hz, $w_0=0.1g$, $n=3$; (**b**) The effect of the number of considered mode shapes in the simulation on the results. Other parameters for simulation are $s_0=0.5/a_r$, $f=90$ Hz, $w_0=0.1g$ (note that the response curve of three-mode case (solid black) coincide with the response curve of the five-mode case (dashed red).

**Figure 5:**
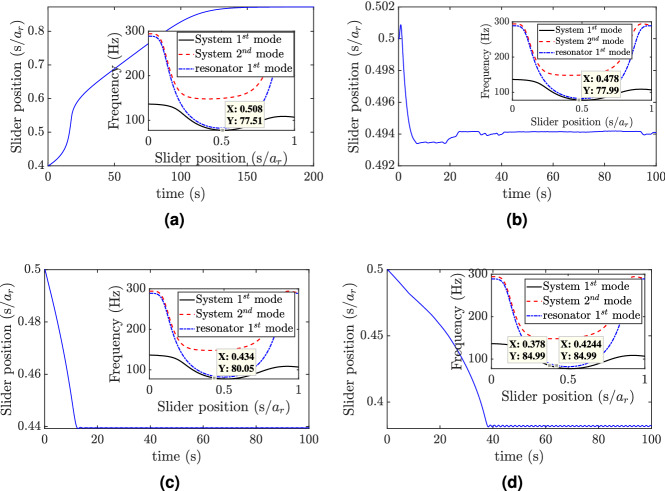
Slider position at different excitation frequencies: (**a**) f = 77 Hz, $w_0=0.1g$; (**b**) f = 78 Hz, $w_0=0.1g$; (**c**) f = 80 Hz, $w_0=0.1g$; (**d**) f = 85 Hz, $w_0=0.1g$.

**Figure 6:**
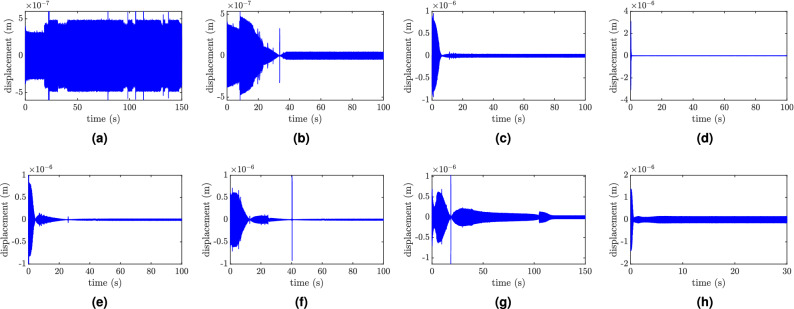
Tip displacement of the primary structure at different excitation frequencies: (**a**) f = 78 Hz, $w_0=0.1g$; (**b**) f = 85 Hz, $w_0=0.1g$; (**c**) f = 130 Hz, $w_0=0.1g$; (**d**) f = 140 Hz, $w_0=0.1g$; (**e**) f = 150 Hz, $w_0=0.1g$; (**f**) f = 155 Hz, $w_0=0.1g$; (**j**) f = 200 Hz, $w_0=0.5g$; (**h**) f = 250 Hz, $w_0=0.8g$.

### Passive self-tunability of the system and its effect on the system response

Based on the system parameters given in Table 1, we numerically integrate the discretized system using the different algorithms discussed in the methods section. In this analysis, we focus on the first resonance mode of the resonator, which ranges from 82.2 to 288.8 Hz.

Firstly, we determine the mode shapes and resonance frequency of the combined structure at the anticipated steady-state position (i.e., the equilibrium position) for a specific excitation frequency. Then, we numerically integrate the system by assuming a fixed mode shape. Secondly, we obtain the instantaneous linear frequencies and mode shapes and save them in a data set. Then, this data set is called in the numerical integration simulations at different excitation frequencies. Finally, we assume that the change in the slider position is slower than the system dynamics. Therefore, we update the mode shapes every 0.1 seconds and use the final state of this slow time step as initial conditions for the next simulation/time-step. The results of these three different methods for the slider position are shown in Fig. 4a. The results indicate that the assumed mode method may fail in determining the steady-state position of the slider accurately. Indeed, the assumed mode method shows that the slider can move to either end of the slot i.e., either $s=0.05a_r$ or $s=0.95a_r$ (more details on the tracking of equilibrium position is discussed in the experimental section). On the other hand, the other algorithms show that the slider settles down almost at the same steady-state position with some discrepancies limited to the transient response. However, the simulation time for the semi-variable modes algorithm is significantly lower as compared to the variable mode method, which updates the instantaneous mode shapes at every time step. Therefore, we will use the semi-variable algorithm in all subsequent simulations in the current study.

In order to determine the sufficient number of mode shapes that yield accurate results, we simulate the system with different numbers of mode shapes and plot the results in Fig. 4b. The results demonstrate that including only one mode shape in the analyses yields inaccurate results as compared to including three modes. However, increasing the number of considered modes beyond three does not significantly affect the simulation results, in most cases, except increasing the computation cost significantly. Thus, we will consider only the nearest three mode shapes from the excitation frequency in further analyses if it is not mentioned otherwise. Yet, including more number of modes in the numerical simulations may yield more accurate results, in some cases, especially at higher excitation frequencies (see the supplementary material for more information).

**Figure 7:**
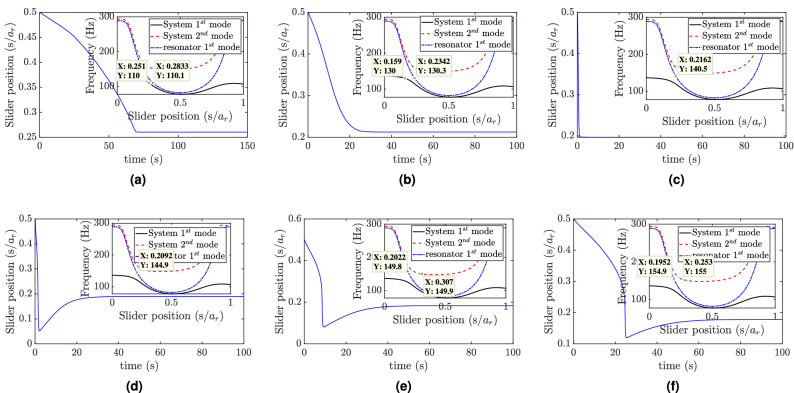
Slider position at different excitation frequencies: (**a**) f = 110 Hz, $w_0=0.1g$; (**b**) f = 130 Hz, $w_0=0.1g$; (**c**) f = 140 Hz, $w_0=0.1g$; (**d**) f = 145 Hz, $w_0=0.1g$; (**e**) f = 150 Hz, $w_0=0.1g$; (**f**) f = 155 Hz, $w_0=0.1g$.

Having established the number of mode shapes required for simulations, next we investigate the slider response by dividing the frequency range of the resonator and the combined structure into three regions. For the low-frequency region, the variations in slider positions for different excitation frequencies are shown in Fig. 5. First, we excite the system at a frequency below the frequency ranges of both the resonator and the combined structure, as shown in Fig. 3b. In this case, the slider does not track any equilibrium position within these frequency ranges; instead, it settles down near the free end of the structure, which represents a vibration anti-node of the structure, as shown in Fig. 5a. Increasing the excitation frequency slightly to a frequency within the range of the combined structure and outside the range of the resonator shows that the slider will track an equilibrium position near the middle of the resonator, as depicted in Fig. 5b, c. It is noteworthy that the frequency does not hit the exact equilibrium position. This is due to numerical error in frequency calculations, where the frequency-position in this region has a small slope that significantly differs the slider position with small change in frequency calculations. This position tunes the structure to resonance instead of the resonator and results in a significant increase in the structure’s tip displacement, as shown in Fig. 6a. Increasing the excitation frequency to 85 Hz, which lies within the resonance frequency range of the resonator, shows that the slider starts to tune itself to the applied excitation frequency, as shown in Fig. 5d. Although the steady-state position of the slider does not precisely coincide with the resonator frequency curve, it neither coincides with the resonance frequency curve of the structure. Thus, the tip displacement will be reduced significantly as the slider reaches its equilibrium position, as shown in Fig. 6b.

**Figure 8:**
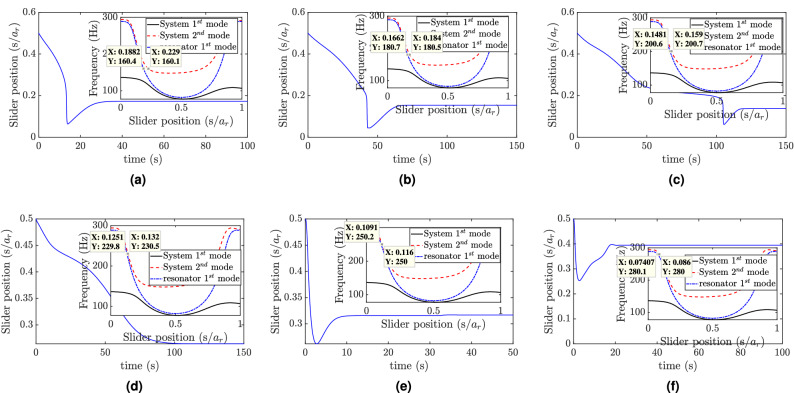
Slider position at different excitation frequencies: (**a**) f = 160 Hz, $w_0=0.2g$; (**b**) f = 180 Hz, $w_0=0.3g$; (**c**) f = 200 Hz, $w_0=0.5g$; (**d**) f = 230 Hz, $w_0=0.8g$; (**e**) f = 250 Hz, $w_0=0.8g$; (**f**) f = 280 Hz, $w_0=0.8g$.

Next, we focus on the frequency region close to the region where the structure does not have any resonance frequency and plot the slider positions and the tip displacements for different excitation frequency, and the results are shown in Figs. 7 and 6c–f, respectively. As the frequency increases toward the region where the slider and the structure frequency-position curves start departing from each other, the slider tracks an equilibrium position which tunes itself to the resonance, as shown in Fig. 7a, b. Note that this position is away from the equilibrium position that tunes the whole structure to resonance. This phenomenon will also lead to a gradual reduction in the tip displacement as the slider reaches its equilibrium position, as depicted in Fig. 6c. The vibration reduction observed in this figure is more significant as compared to the low-frequency region, as shown in Fig. 6b. In Fig. 7, as we move the excitation frequency inside the region where only the resonator has resonance frequencies, we observe that the slider tracks the equilibrium positions which tunes it to the applied excitation frequency. This observation is also supported by the absence of any resonance frequency for the combined structure for all possible slider positions in this region. Inside this region, the tip displacement becomes significantly low when the slider reaches the equilibrium position as compared to the response at other slider location, as can be seen in Fig. 6c. As the excitation frequency increases above this range (see Fig. 7e, f), where the structure’s second vibration mode does have resonance frequency along with the resonator, the slider still tracks an equilibrium position that tunes itself to the excitation frequency rather than the combined structure resonance frequency. However, the steady-state displacement of the system gradually increases at the equilibrium position as compared to other slider positions and the results in the low-frequency region (see Fig. 6 e, f).

Finally, we excite the structure at frequencies within the region where the slider and combined structure frequency-position curves are closer and the results are shown in Figs. 8 and 6g, h. In this region, the slider still tracks equilibrium positions that tune it to the excitation frequency, as shown in Fig. 8a, b. However, Fig. 8c demonstrates that the frequencies of the resonator and the combined structure become closer by increasing the excitation frequency to around 230 Hz. Yet the slider equilibrium position is closer to tune the resonator rather than the combined structure. This further leads to reducing the vibration attenuation recorded at the tip as the slider moves along the slot, as depicted in Fig. 6g. Indeed, this increase in the steady-state response increases with the increase in the excitation frequency in this region. With further increase in the excitation (i.e., above 230 Hz), the slider does not track an equilibrium position anymore. Indeed, Fig. 8e, f indicates that the slider equilibrium position does not match the anticipated position highlighted in the small windows. Moreover, the slider’s equilibrium position approaches the mid slot point, which might be close to an anti-node of higher vibration modes. This mistuning yields a significant increase in the steady-state response as compared with the slider at other locations, as shown in Fig. 6h.

### Experimental validation

**Figure 9:**
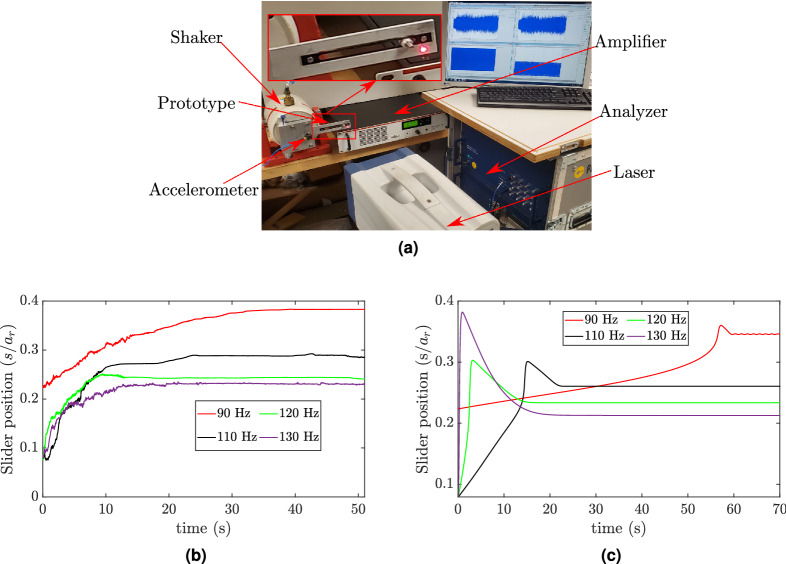
(**a**) Key components of the experimental setup; (**b**) Measured slider position at different excitation frequencies; (**c**) Analytical slider position at different excitation frequencies.

In order to verify the analytical observations, we conduct an experiment on a prototype representing the proposed structure. This prototype with its experimental setup are shown in Fig. 9a, and its dimensions are given in Table 1. It is noteworthy that we changed the total length of the primary structure to $a=0.131$ m instead of the values given in Table 1. This change in length should shift the structure’s frequencies up. As we recall from our analytical analyses, we neglected several parameters in our calculations. These parameters include the slot where the slider moves and the slider dimensions. However, we emphasize that these parameters should not significantly affect our qualitative study.

**Figure 10:**
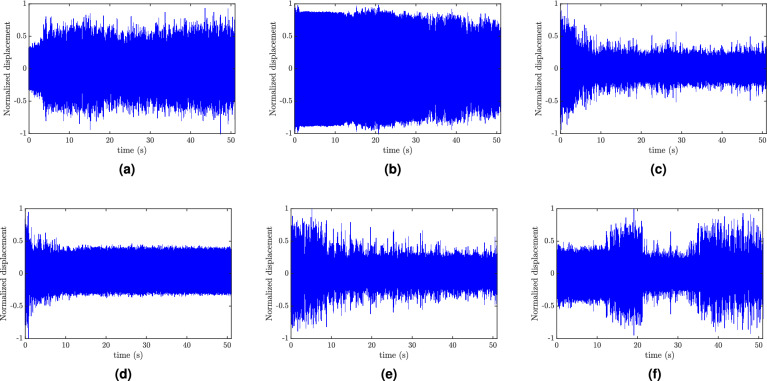
Test measurement of the tip displacement: (**a**) f = 230 Hz, $s_{start}=0.8a_r$, $s_{end}=0.9a_r$, $w_0=6g$; (**b**) f = 145 Hz, $s_{start}=0.74a_r$, $s_{end}=0.86a_r$, $w_0=0.6g$; (**c**) f = 100 Hz, $s_{start}=a_r$, $s_{end}=0.8a_r$, $w_0=1.37g$; (**d**) f = 70 Hz, $s_{start}=82a_r$, $s_{end}=0.66a_r$, $w_0=1.44g$; (**e**) f = 60 Hz, $s_{start}=0.77a_r$, $s_{end}=0.52a_r$, $w_0=1g$; (**f**) f = 55 Hz, $s_{start}=0.77a_r$, $s_{end}=0.6a_r$, $w_0=1g$.

First, we record the slider displacement, which is free to move along the slot, by applying a sinusoidal signal with different frequencies and placing the slider at different initial positions. Note that we focus on demonstrating the qualitative analytical observations without paying attention to the exact quantitative part. This is because our analytical investigation neglected some design parameters (see supplementary material) and the effect of friction, which is beyond the scope of the current analysis. Indeed, the analytical model of the friction force requires considering the contact laws and contact kinematics as discussed in^19^. However, neglecting these parameters should not affect the qualitative match between the analytical and experimental observations. The slider position is measured from its starting position till it reaches the equilibrium position. These measurements are shown in Fig. 9b for different excitation frequencies. For the low-frequency region, we conducted a test at 90 Hz. The results show that the slider settles down at the equilibrium position and stays there as time evolves, as can be seen in Fig. 9b. Indeed, this new equilibrium position tunes the resonator to the applied excitation frequency and should result in significant decrease in the tip displacement (this observation is supported by experimental results for the tip displacement shown in Fig. 10). These results show a qualitative analogy with the analytical results shown in Figs. 5d and 6b. Next, we examine the middle-frequency region by exciting the system at 110 Hz, 120 Hz, and 130 Hz. With increasing frequency, the slider tends to track an equilibrium position closer to the fixed end. This observation also shows a good agreement with the analytical results. On the other hand, for the frequencies that are used in the test, we plot the counterpart analytical slider positions in Fig. 9c. The results further illustrate the qualitative similarities between the analytical and experimental results since the slider at each excitation frequency tracks almost the same experimental equilibrium position, which changes with changing excitation frequency. However, these equilibrium positions are shifted down as compared to the experimental equilibrium positions due to neglecting some design parameters in the analytical model (see supplementary material). Moreover, to reach the equilibrium and steady state position, there are some discrepancies between the analytical and experimental results shown in Fig. 9b, c. These discrepancies may be attributed to the adaptive algorithm that is used in the numerical simulation (see the method section) or the underestimation of the analytical damping model.

Next, we report the the experimental time response of the tip displacement for various parameters in Fig. 10, where $s_{start}$ is the initial position of the slider and $s_{end}$ is the equilibrium position of the slider. For high-frequency region (Fig. 10a), the results indicate that the tip displacement increases as the slider reaches its equilibrium position. This observation can be justified as frequencies of both the resonators and the structure are close to each other in this region (see Fig. 3b). Therefore, the slider keeps hitting the resonance frequency of the structure as it oscillates around its equilibrium position ( $s_{end}=0.9a_r$ in the case at 230 Hz), leading to high amplitude oscillations of the tip. This observation shows a good agreement with the analytical observation reported in Fig. 6h. However, for regions where the frequencies of the slider and the structure are away from each other, one can observe a reduction in the response as the slider settles down at its equilibrium position, as shown in Fig. 10b for excitation frequency equal to 145 Hz. We emphasize that the recorded reduction is not significant since the slider in this case approaches its equilibrium position from the left (its initial position is to the left from the equilibrium position as depicted in Fig. 3b). Therefore, it does not hit any resonance frequency of the structure on its way. Moreover, the response for this initial position is lower. On the other hand, a significant reduction can be observed in the tip displacement as the slider approaches its equilibrium position from the right side, as shown in Fig. 10c, d. By lowering the excitation frequency to 60 Hz (Fig. 10e), the slider tracks the middle point as an equilibrium position, indicating that the minimum frequency of the resonator obtained experimentally is around 60 Hz. At this frequency, a significant reduction can also be observed in the tip displacement as the slider settles down at its equilibrium position. Further, it can be observed from Fig. 10a–e that the final equilibrium position of the slider tends to move from $0.9a_r$ to $0.5a_r$ as we reduce the excitation frequency from 230 to 60 Hz. This observation is similar to the experimental observations shown in Fig. 9b and the analytical observations shown in Figs. 5, 7, and 8. In addition, the observed reduction in the structure displacement as the slider tunes the resonator corroborates our analytical observations about the self-tuning capability of the proposed resonator, which was demonstrated in Fig. 6a, b. Finally, we show the tip displacement of the system at frequency below the resonator frequency range (i.e., 55 Hz) in Fig. 10f. The results show that the system response is not attenuated at all times and slider positions. In addition, the slider tracks an unanticipated equilibrium point. These two observations also show a good agreement with the analytical results in Figs. 5a and 6a.

## Discussion

**Figure 11:**
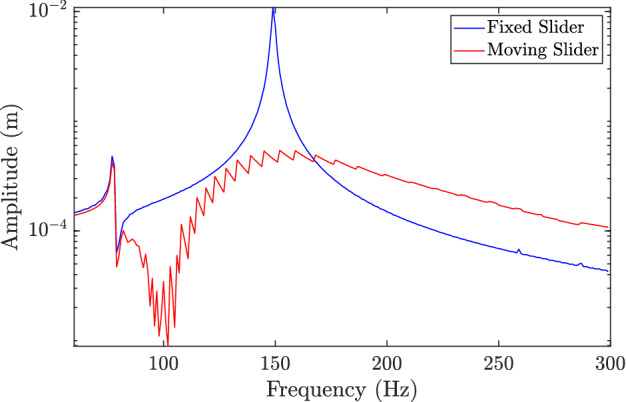
FRF comparison between the self-tuning resonator and the fixed resonator at 0.5$s/a_r$.

The described analytical and experimental investigations demonstrate the ability of the proposed self-tuning resonator to tune itself to the excitation frequency over a wide range of frequencies. This range can be controlled by optimizing the resonator and structure design. For instance, increasing the effective mass of the resonator can further split the resonance frequency modes of the structure. However, our analyses showed the presence of some frequencies, in particular very low and high-frequency regions (defined in the previous section), where the resonator may not be able to tune itself. Therefore, integrating the current passive system with an external actuator, which forces the resonator to perfectly tune itself and reduces its oscillation at the equilibrium position, may significantly widen the frequency band that can be controlled by this resonator. Assuming that the resonator can always tune itself to its equilibrium position (i.e., in perfect operation conditions), we determine the steady-state response of the proposed structure and compare it to the fixed frequency resonator. These FRFs are shown in Fig. 11. The results demonstrate the superiority of the self-tuning resonator as compared to the fixed frequency resonator in terms of structure response at a wide range of frequencies. Moreover, no sharp high amplitude peak can be observed within the target frequency. Finally, the present study is focused on the first mode resonance frequency range of the resonator; however, higher frequency modes are also worthy of investigation given that some of them can stretch over several hundred of hertz. Again, this range may be controlled by changing the design parameters.

## Methods

### Adaptive algorithm to update the system spatial status

The spatial parameters (i.e., the mode shapes and frequencies) need to be imposed in the system before integrating the system numerically over time. However, as the slider moves along the beam, the spatial parameters will change. Hence, assuming a fixed value may result in erroneous results. Therefore, we need to update the value of the spatial parameters to adapt the instantaneous slider position as the integration time evolves. To overcome this problem, one can determine the spatial parameters at each time step based on the instantaneous slider position inside an anonymous function that is called by MATLAB solver. Then, the calculated parameters will be fed into the state-space model and integrated numerically. However, this algorithm is computationally expensive since it needs to determine the system’s natural frequency at each step. For a simple system with only one resonator, one can evaluate all possible frequencies and mode shapes by sweeping the slider position over the resonator with a small step and save the obtained data set prior to simulation. In addition, obtaining the data set can be done by parallel computation using multiple nodes. Since the slider may pass the same position at different time steps, determining the spatial parameter at each frequency step is computationally expensive. Instead, the data set can simply be called once for all time steps and the spatial parameters can be picked based on the instantaneous slider position from the previously obtained data set. The latter approach can reduce the computation time significantly for this simple case; however, it may not be practical to obtain the data set when we have multiple self-tuning resonator on the structure. Indeed, the size of the data set will be increased dramatically for the case of multiple resonators.

Another algorithm that can further reduce the computational effort can be realized by updating the spatial parameters at a time step larger than the simulation time step. For instance, if we run a simulation for 100 second with a time step 0.001 second (please note that the time step in MATLAB built-in integrator is adaptive, and it can be much smaller than this to get the simulation converging), we update the spatial parameters every 0.1 seconds. This assumption can be valid since the dynamic of the slider is usually slower than the dynamic of the system. Therefore, the simulation cost can be further reduced regardless of the algorithm used in the previous paragraph. Although this algorithm affects the accuracy of the transient response, it neither affects the equilibrium slider position nor the steady-state response. The effect on the accuracy in the transient response may appear as spikes at specific time instants. These spikes are attributed from numerical artifacts in the transient response due to the sudden change in the mode shapes values as the slider tracks its equilibrium position. In addition, these spikes may result from hitting the combined system resonance frequency for a short time as the slider moves along its track.

### Experimental setup

The experimental setup of the proposed structure is shown in Fig. 9. The structure is excited by an electromagnetic shaker (LDS V408). This excitation is represented by a base excitation. The structure is fixed to the shaker from its fixed end using a fixture with a resonance frequency much higher than the investigated frequency range. The applied base excitation is measured by an accelerometer (PCB 356A16) fixed at the fixture. To avoid any mass addition on the free end of the structure, which may change the system frequency, we use a Polytec Laser Doppler Vibrometer (Polytec PSV-500) to record the structure response. The laser beam is pointed at the tip of the structure’s free end. The velocity of the slider is also measured using Polytec Laser Doppler Vibrometer (Polytec PSV-500). This signal is further integrated to obtain the displacement of the slider by applying a low pass filter that avoids error build-up in the integration due to noise. The signal generated by these sensors is recorded and analyzed using (Polytec DAQ). The DAQ is also used to generate the input profile which is amplified using an amplifier (LPA100) that drives the shaker.

## Supplementary Information

Supplementary Information.
