# Supplementary material for: Towards a self tuning sliding mass metastructure
Source: Sci Rep. 2021 Nov 3;11:21630. doi: 10.1038/s41598-021-00526-w (PMC8566453; doi:10.1038/s41598-021-00526-w)
Supplement: Supplementary file 1 — Supplementary Information. [file 41598_2021_526_MOESM1_ESM.pdf]

Supplementary materials: Towards a self-tuning sliding-mass metastructure

Mohammad A Bukhari[1] **and Oumar R Barry**[1,*]

[1]Virginia Tech, Mechanical Engineering, Blacksburg, 24060, USA
[*]obarry@vt.edu

ABSTRACT

1 Mathematical modelling

For the proposed structure shown in Fig. 1 in the manuscript, the governing equations of motion of the structure, the self-tuning resonator, and the slider can be expressed as:

$$m_m \ddot{\bar{w}}_m + EI_m \bar{w}_m'''' = -m_m \ddot{\bar{w}}_0 \tag{1}$$

$$m\ddot{\bar{w}}_r + EI_r \bar{w}_r'''' + M[\ddot{\bar{w}}_r + 2\dot{\bar{s}}\dot{\bar{w}}_r' + \ddot{\bar{s}}\bar{w}_r' + \dot{\bar{s}}^2 \bar{w}_r'']_{x=\bar{s}} = -m_r \ddot{\bar{w}}_0 - M\ddot{\bar{w}}_0 \delta(x-\bar{s}) \tag{2}$$

$$M\ddot{\bar{s}} + M\bar{w}_r'[\ddot{\bar{w}}_r + 2\dot{\bar{s}}\dot{\bar{w}}_r' + \ddot{\bar{s}}\bar{w}_r' + \dot{\bar{s}}^2 \bar{w}_r'']_{x=\bar{s}} = -M\bar{w}_r'\ddot{\bar{w}}_0 \delta(x-\bar{s}) \tag{3}$$

where $\bar{w}_m$ is the displacement of the primary structure, $\bar{w}_r$ is the displacement of the resonator, $\bar{s}$ is the displacement of the slider, δ is the Dirac delta function, $(\dot{})$ is the derivative with respect to time, and $(')$ is the derivative with respect to space. Note that the flexural rigidity EI_m, EI_r and the mass per unit length m_m, m_r vary along the structure. Further discussion on the flexural rigidity and the mass per unit length will be presented in Section 2. To normalize the system, the following dimensionless parameters are introduced

$$\omega_{nm}^2 = \frac{EI_m}{m_m a^4}, \omega_{nr}^2 = \frac{EI_r}{m_r a^4}, M_r = \frac{M}{m_r}, w_m = \bar{w}_m/a, w_r = \bar{w}_r/a, s = \bar{s}/a, \zeta = \bar{x}/a, \tau = \frac{t}{a^2}\sqrt{\frac{E_m}{m_m}},. \tag{4}$$

substituting Eq. 4 into Eqs. 1-3 yields the following normalized equations

$$\ddot{w}_m + w_m'''' = -\ddot{w}_0 \tag{5}$$

$$\ddot{w}_r + \frac{\omega_{nr}^2}{\omega_{nm}^2} w_r'''' + M_r[\ddot{w}_r + 2\dot{s}\dot{w}_r' + \ddot{s}w_r' + \dot{s}^2 w_r'']_{\zeta=s} = -\ddot{w}_0 - M_r \ddot{w}_0 \delta(\zeta - s) \tag{6}$$

$$\ddot{s} + w_r'[\ddot{w}_r + 2\dot{s}\dot{w}_r' + \ddot{s}w_r' + \dot{s}^2 w_r'']_{\zeta=s} = -w_r'\ddot{w}_0 \delta(\zeta - s) \tag{7}$$

1.1 Galerkin's projection

In order to simulate the system numerically, we discretize the partial differential equation given in Eqs. 5-6 into a system of ordinary differential equations. This can be done by Galerkin's projection, which further requires writing the solution of w_m, w_r as

$$w_m = \sum_{n=1}^{\infty} \phi_{nm}(\zeta) A_n(\tau) \tag{8}$$

$$w_r = \sum_{n=1}^{\infty} \phi_{nr}(\zeta) A_n(\tau) \tag{9}$$

where $A_n(\tau)$ is an unknown function of time for the nth mode, $\phi_{nm}(\zeta)$ is the nth mode shape of the primary structure, and $\phi_{nm}(\zeta)$ is the nth mode shape of the resonator. Substituting Eqs. 8-9 in Eqs. 5-6 yields

$$\sum_{n=1}^{\infty} \phi_{nm}(\zeta)\ddot{A}_n(\tau) + \sum_{n=1}^{\infty} \phi_{nm}^{iv}(\zeta)A_n(\tau) = -\ddot{w}_0 \tag{10}$$

$$\begin{gathered}\sum_{n=1}^{\infty} \phi_{nr}(\zeta)\ddot{A}_n(\tau) + \frac{\omega_{nr}^2}{\omega_{nm}^2}\sum_{n=1}^{\infty} \phi_{nr}^{iv}(\zeta)A_n(\tau) + M_r[\sum_{n=1}^{\infty} \phi_{nr}(\zeta)\ddot{A}_n(\tau) + 2\dot{s}\sum_{n=1}^{\infty} \phi'_{nr}(\zeta)\dot{A}_n(\tau) + \\ \ddot{s}\sum_{n=1}^{\infty} \phi'_{nr}(\zeta)A_n(\tau) + \dot{s}^2\sum_{n=1}^{\infty} \phi''_{nr}(\zeta)A_n(\tau)]_{\zeta=s} = -\ddot{w}_0 - m_r\ddot{w}_0\delta(\zeta - s)\end{gathered} \tag{11}$$

$$\begin{gathered}\ddot{s} + \sum_{n=1}^{\infty} \phi'_{nr}(\zeta)A_n(\tau)[\sum_{n=1}^{\infty} \phi_{nr}(\zeta)\ddot{A}_n(\tau) + 2\dot{s}\sum_{n=1}^{\infty} \phi'_{nr}(\zeta)\dot{A}_n(\tau) + \ddot{s}\sum_{n=1}^{\infty} \phi'_{nr}(\zeta)A_n(\tau) + \\ \dot{s}^2\sum_{n=1}^{\infty} \phi''_{nr}(\zeta)A_n(\tau)]_{\zeta=s} = -\sum_{n=1}^{\infty} \phi'_{nr}(\zeta)A_n(\tau)\ddot{w}_0\delta(\zeta - s)\end{gathered} \tag{12}$$

The reduced order equations can be obtained by multiplying Eq. 10 by $\phi_{pm}(\zeta)$ and Eq. 11 by $\phi_{pr}(\zeta)$, adding them together, integrating over the normalized length, and recalling the orthogonality conditions as

$$\begin{gathered}\ddot{A}_p + \omega_p^2 A_p + M_r\phi_p(s)[\sum_{n=1}^{\infty} \phi_n(s)\ddot{A}_n(t) + 2\dot{s}\sum_{n=1}^{\infty} \phi'_n(s)\dot{A}_n(t) + \ddot{s}\sum_{n=1}^{\infty} \phi'_n(s)A_n(t) + \\ \dot{s}^2\sum_{n=1}^{\infty} \phi''_n(s)A_n(t)] = -\int_0^1 \phi_p(\zeta)\ddot{w}_0 d\zeta + m_1\phi_p(s)\ddot{w}_0\end{gathered} \tag{13}$$

where $\ddot{w}_0 = w_0 \sin(2\pi f t)$, and ϕ_r, ϕ_p are the rth and the pth mode shape of the combined structure (i.e., the primary structure and the resonator), respectively.

After discretizing the system, the state space form of Eqs. 12-13 can be solved numerically in MATLAB. The resulting state-space model has a dimension $(2r+2)$, where r is the number of modes considered in the simulation. It is noteworthy here that the system is in an algebraic loop since each derivative is a function of the other derivatives. In order to solve this system, we need to break the loop by building a matrix for the coefficients of derivatives on the left hand of the state-space model, then multiplying the state-space model by the inverse of this matrix. Further, linear viscous damping is added to Eqs. 12-13 in the numerical simulation to achieve better convergence.

2 Adaptive linear mode shapes algorithm to simulate the system

In order to simulate the state-space model discussed in the above section, we obtain the space component (i.e., mode shapes) to integrate over the time only. The conventional approach to solve the current problem is to employ the mode shapes of the bare beam. This method is known as the assumed mode method and the associated error can be small if the mass of attachment is small as compared to the primary beam. However, the error becomes significant for larger masses and hence, exact mode shapes and orthogonality conditions need to be considered (1–3). In the current study, the slider moves along the resonator till it reaches an equilibrium position. Therefore, the mode shapes and the resonance frequency of the system continuously change as the slider moves. Assuming a fixed mode shape throughout the numerical simulation may lead to erroneous results and the slider may not settle down at the correct equilibrium position that tunes the resonator to the excitation frequency.

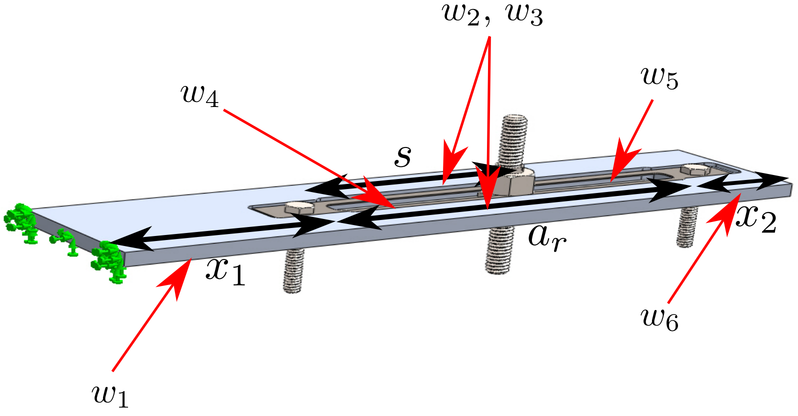

Figure 1. System portions used in determining the system instantaneous mode shapes.

To obtain the instantaneous exact linear mode shapes (i.e., at the instantaneous slider position) of the combined structure, the linear equation of motion corresponding to the free vibration for each portion of the system can be written as

$$\ddot{w}_i + \omega_i^2 w_i'''' = 0, \quad i = 1, 2, 3...6. \tag{14}$$

with

$$\omega_i^2 = \frac{E_i I_i}{m_i L_i^4} \tag{15}$$

where E_i is the modulus of elasticity, I_i is the second moment of area, m_i is the mass per unit length, and L_i is the length of the ith portion of the structure. To determine the system natural frequency, we divide the structure into 6 portions as shown in Fig. 1. The first portion stretches from the fixed end of the main structure to the first fixed point of the resonator. Therefore, it has a length of $L_1 = x_1$, modulus of elasticity $E_1 = E_m$, mass per unit length $m_1 = \rho_m t_m w_m$, and $I_1 = 1/12 t_m^3 w_m$. The second and the third portions of the system stretch parallel to the resonator along both sides on the main structure. These two portions will be identical and will have $L_2 = L_3 = a_r$, $E_2 = E_3 = E_m$, $m_2 = m_3 = \rho_m t_m w_{m2}$, and $I_2 = I_3 = 1/12 t_m^3 w_{m2}$, where w_{m2} is the width of each portion. The fourth and the fifth portions represent the resonator beam and they are divided by the instantaneous slider position s. Therefore, they both have $E_4 = E_5 = E_r$, $m_4 = m_5 = \rho_r t_r w_r$, and $I_4 = I_5 = 1/12 t_r^3 w_r$. However, their length depends on the instantaneous slider position, such that $L_4 = s$ and $L_5 = a_r - s$. In order to simplify the model of the resonator, we neglect the slot in which the slider moves. Finally, the last portion of the system stretches from the end of the resonator till the end of the structure beam with $E_6 = E_m$, $L_6 = x_2$, $m_6 = \rho_m t_m w_m$, and $I_6 = 1/12 t_m^3 w_m$.

2.1 Instantaneous linear mode shapes and frequencies

At each instantaneous slider position, we calculate the linear mode shapes and resonance frequencies by solving for the boundary and continuity conditions (i.e., in this case, we have 4×6=24 conditions). At the fixed end, the two boundary conditions can be defined as

$$w_1(0,\tau) = w_1'(0,\tau) = 0, \tag{16}$$

while at the free end, the other two boundary conditions can be expressed as

$$w_6''(1,\tau) = w_6'''(1,\tau) = 0. \tag{17}$$

The first set of the continuity conditions are defined at x_1 from the fixed end. In particular, these continuity conditions are defined between the end of the first portion (i.e., at x_1) and the beginning of the second, third, and fourth portion. These continuity conditions are

$$w_1(x_1,\tau) = w_2(x_1,\tau) = w_3(x_1,\tau) = w_4(x_1,\tau), \tag{18}$$

$$w'_1(x_1,\tau) = w'_2(x_1,\tau) = w'_3(x_1,\tau) = w'_4(x_1,\tau), \tag{19}$$

$$E_1 I_1 w''_1(x_1,\tau) = E_2 I_2 w''_2(x_1,\tau) + E_3 I_3 w''_3(x_1,\tau) + E_4 I_4 w''_4(x_1,\tau), \tag{20}$$

$$E_1 I_1 w'''_1(x_1,\tau) = E_2 I_2 w'''_2(x_1,\tau) + E_3 I_3 w'''_3(x_1,\tau) + E_4 I_4 w'''_4(x_1,\tau). \tag{21}$$

The second set of continuity conditions are defined between the fourth and the fifth portion at the slider position (i.e., $x_1 + s$). These conditions are

$$w_4(x_1+s,\tau) = w_5(x_1+s,\tau), \tag{22}$$

$$w'_4(x_1+s,\tau) = w'_5(x_1+s,\tau), \tag{23}$$

$$w''_4(x_1+s,\tau) = w''_5(x_1+s,\tau), \tag{24}$$

$$E_4 I_4 w'''_4(x_1+s,\tau) = E_5 I_5 w'''_5(x_1+s,\tau) + m_r \ddot{w}_4(x_1+s,\tau). \tag{25}$$

Finally, the last set of continuity conditions are defined at the end of the resonator beam (i.e., $x_1 + a_r$). Particularly, between the beginning of the sixth portion and the end of second, third, and fifth portion. The conditions are

$$w_6(x_1+a_r,\tau) = w_2(x_1+a_r,\tau) = w_3(x_1+a_r,\tau) = w_5(x_1+a_r,\tau), \tag{26}$$

$$w'_6(x_1+a_r,\tau) = w'_2(x_1+a_r,\tau) = w'_3(x_1+a_r,\tau) = w'_5(x_1+a_r,\tau), \tag{27}$$

$$E_6 I_6 w''_6(x_1+a_r,\tau) = E_2 I_2 w''_2(x_1+a_r,\tau) + E_3 I_3 w''_3(x_1+a_r,\tau) + E_5 I_5 w''_5(x_1+a_r,\tau), \tag{28}$$

$$E_6 I_6 w'''_6(x_1+a_r,\tau) = E_2 I_2 w'''_2(x_1+a_r,\tau) + E_3 I_3 w'''_3(x_1+a_r,\tau) + E_5 I_5 w'''_5(x_1+a_r,\tau). \tag{29}$$

Since the aim is to determine the instantaneous linear mode shapes, the solution of each portion can be written as

$$w_i = X_i(\zeta) e^{j\omega\tau} \tag{30}$$

where $X_i(\zeta)$ is the linear mode shape and can be written as

$$X_i(\zeta) = A_i \sin(\beta_i \zeta) + B_i \cos(\beta_i \zeta) + C_i \sinh(\beta_i \zeta) + D_i \cosh(\beta_i \zeta) \tag{31}$$

where $\beta_i = \sqrt{\omega_i \omega}$.

Upon imposing Eqs. 30-31 into Eqs. 16-29, the instantaneous mode shapes and frequencies of the system can be determined by solving the resulting system.

Table 1. Resonance frequencies for the primary structure and the resonator obtained by SolidWorks.

Position s/a_r	Structure 1st mode (Hz)		Structure 2nd mode (Hz)		Resonator 1st mode (Hz)	
	SolidWorks	Analytical	SolidWorks	Analytical	SolidWorks	Analytical
0.5	70.65	78.15	150.64	177	72.68	82.2
0.33	74.07	92.97	150.49	176	75.75	97.11
0.25	84.20	113.2	150.04	180.8	86.07	123.1
0.19	114.6	135.1	151.28	195.6	118.65	158.8
0	145.11	157	377.84	298.3	377.04	288.7

3 Frequency calculations using SolidWorks

To study the effect of these parameters, we conduct frequency analyses using SolidWorks and report the resonance frequencies of the structure and the resonator only at different slider positions. The obtained resonance frequencies from both analytical simulations and Solidworks are tabulated in Table 1. Although neglecting the resonator parameters can cause some errors in both the structure and the resonator frequencies, the results show similar behavior in the frequency trend for both the structure and the resonator. In particular, the first resonance frequency increases with moving the slider toward the fixed end inside the slot, where it ranges from 70.65 Hz to 145.11 Hz. In addition, the second mode frequency also follows the same trend, where it ranges from 150.64 Hz to 377.84 Hz. It can be observed that the values of the second resonance frequency for the slider position between 0.25-0.5 a_r/a are close to each other. This observation can be explained by the plateau curve in this region, as it was observed in (Fig. 3(b) in the main manuscript), in addition to numerical errors in simulations. In general, the upper limit of the frequencies for different modes went up due to a reduction in the length of the primary structure, while the lower limit went down due to an increase in the mass ratio of the slider to the resonator as we remove the slotted mass and include the dimension of the slider in the simulation. On the other hand, the frequency region where the structure does not have any resonance frequencies (i.e., between the first and the second modes) was also reduced due to the reduction in the effective mass of the resonator by eliminating the slotted mass. However, the qualitative trends stayed similar to the analytical model.

4 The effect of initial slider position on the system behavior

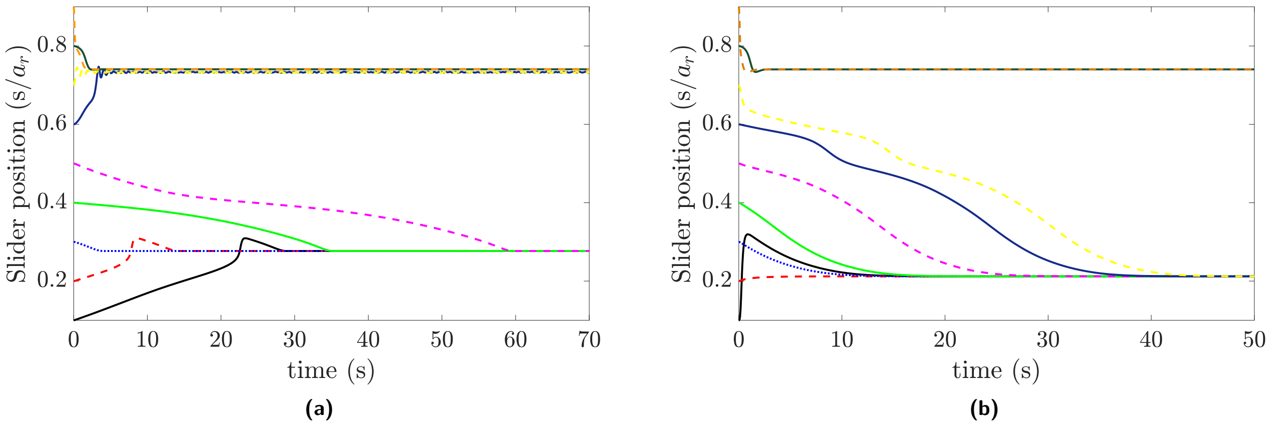

Figure 2. Slider position for different initial positions: (a) f=105 Hz, $w_0 = 0.1g$, 8 modes used in the numerical simulation; (b) f=130 Hz, $w_0 = 0.1g$, 8 modes used in the numerical simulation.

To investigate the effect of the initial position of the slider on the system's behavior, we plot the slider response for different initial positions in Fig. 2. The results demonstrate that the slider tracks different equilibrium positions depending on the initial position of the slider. In particular, there are two equilibrium positions that tune the resonator to the applied excitation frequency, as shown in the main manuscript (Fig.2). The slider tends to track

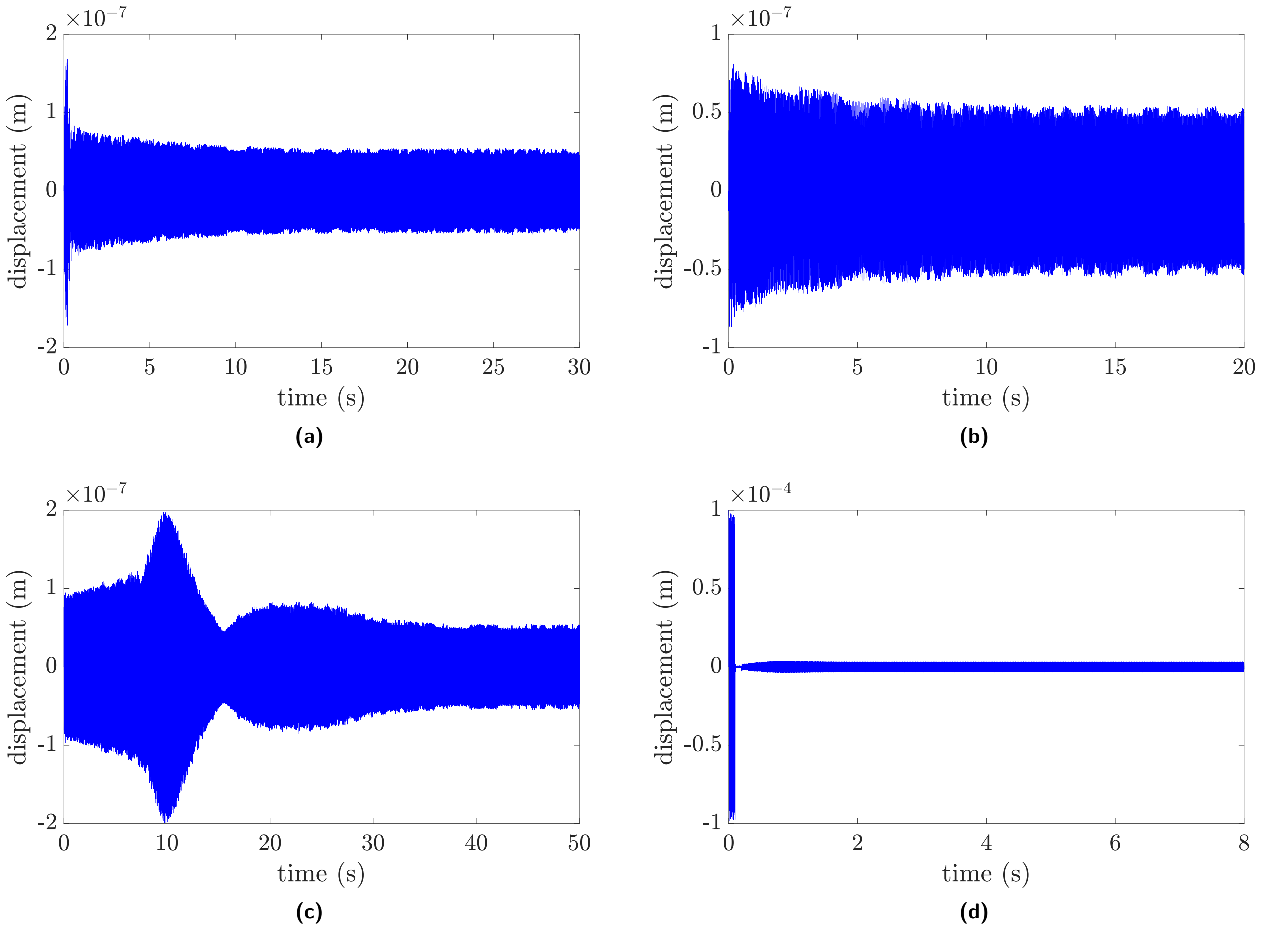

Figure 3. Tip displacement at f=130, $w_0 = 0.1g$, for different initial slider positions: (a) $s_0/a_r = 0.1$, 8 modes used in the numerical simulation; (b) $s_0/a_r = 0.3$, 8 modes used in the numerical simulation; (c) $s_0/a_r = 0.6$, 8 modes used in the numerical simulation; (d) $s_0/a_r = 0.9$, 8 modes used in the numerical simulation.

the closest equilibrium position to tune the resonator. For instance, at excitation frequency f=105 Hz (Fig. 2(a)), the slider tracks an equilibrium position closer to the fixed end for initial positions $s_0/a_r \leq 0.5$. However, the slider tends to settle down near the other equilibrium position for certain initial positions. Indeed, the basins of attractions (equilibrium positions) of this problem are not always symmetric around $s_0/a_r = 0.5$, as shown in Fig. 2(b). This might be attributed to the irregular boundary conditions of the studied system (i.e., the combined system has a fixed end and free end). It is noteworthy that the required transient time to reach the equilibrium position decreases as the distance between the initial position and equilibrium point becomes closer.

Although the slider may track two different equilibrium positions, the tip displacement of the combined system is reduced significantly once the slider reaches any of the equilibrium positions regardless of the initial position, as shown in Fig. 3. However, the initial position of the slider plays a role in the severity of vibrations recorded in the transient response at the tip of the combined structure. For instance, when the initial slider's position $s_0/a_r = 0.1$ (see Fig. 3(a)), the tip displacement is higher at the beginning of the simulation, where the slider is away from the equilibrium position. The tip displacement reduces gradually as the slider get closer to its equilibrium position. However, when the initial position of the slider is closer to the equilibrium point $s_0/a_r = 0.3$ (see Fig. 3(b)), the severity of the oscillations is small in the transient regime. This is because the slider does not meet any position that significantly mistunes the resonator from the excitation frequency as compared to farther initial positions.

References

1. Hassanpour, P., Cleghorn, W., Mills, J. & Esmailzadeh, E. Exact solution of the oscillatory behavior under axial force of a beam with a concentrated mass within its interval. *J. Vib. Control.* **13**, 1723–1739 (2007).

2. Barry, O., Oguamanam, D. & Zu, J. On the dynamic analysis of a beam carrying multiple mass-spring-mass-damper system. *Shock. Vib.* **2014** (2014).

3. Bukhari, M. & Barry, O. Exact nonlinear dynamic analysis of a beam with a nonlinear vibration absorber and with various boundary conditions. *J. Comput. Nonlinear Dyn.* **15** (2020).
